# Femoral lengthening might impair physical function and lead to structural changes in adjacent joints: 10 patients with 27 to 34 years’ follow-up

**DOI:** 10.1080/17453674.2020.1866864

**Published:** 2021-01-07

**Authors:** Patrick A Bjørge, Anne-Therese Tveter, Harald Steen, Ragnhild Gunderson, Joachim Horn

**Affiliations:** a Department of Physiotherapy, Oslo University Hospital, Rikshospitalet, Oslo;; b National Resource Center for Rehabilitation in Rheumatology, Diakonhjemmet Hospital, Oslo;; c Biomechanics Laboratory, Oslo University Hospital, Rikshospitalet, Oslo;; d Department of Radiology, Oslo University Hospital, Rikshospitalet, Oslo;; e Department of Children´s Orthopaedics and Reconstructive Surgery, Oslo University Hospital, Rikshospitalet, Oslo;;; f Institute of Clinical Medicine, Faculty of Medicine, University of Oslo, Oslo, Norway.

## Abstract

Background and purpose — Literature describing long-term functional outcome and osteoarthritis (OA) in adjacent joints after femoral lengthening is rare. We evaluated physical function and the presence of radiographic OA in adjacent joints in 10 patients ≥ 27 years after femoral lengthening.

Patients and methods — We conducted a cross-sectional study of 10 patients treated by unilateral femoral lengthening. Follow-up was between 27 and 34 years. Physical function was evaluated by the 30-second sit-to-stand (30sSTS) and a stair test and was compared with reference values. 4 single-legged hop tests were used to assess difference in physical function between the lengthened and contralateral limb. Radiographic OA was evaluated by joint space width (JSW) and Kellgren and Lawrence (KL) classification.

Results — The patients scored worse compared with reference values on the 30sSTS and stair test, and worse on the lengthened limb on the single- and triple-hop test. Radiographic OA was found in the hip or knee in the lengthened limb in 3 of 10 patients based on JSW and 4 of 10 based on KL. No radiographic OA was found in unlengthened limbs.

Interpretation — Our results showed impaired physical function both in general and of the lengthened limb. Additionally, we found a possible association between femoral lengthening and radiographic OA in adjacent joints in the long term. However, the sample size of the current study is small.

Limb lengthening by the callotasis technique is a well-established method for treatment of leg length discrepancy (LLD). However, literature describing the long-term functional outcome and eventual late side effects such as osteoarthritis (OA) in adjacent joints is rare. Previous studies have investigated whether femoral lengthening impacts muscle strength in the lengthened limb, but results are inconsistent (Bhave et al. 2017, Krieg et al. 2018). Concerns have been raised regarding whether limb lengthening might lead to OA in adjacent joints (Herring 2008, Sneppen et al. 2014). However, to our knowledge there is only 1 published article describing articular damage after femoral lengthening by the callotasis technique. In this animal study, Stanitski (1994) found cartilage injury in the knees of all included canines after completing 30% distraction of initial femoral length. Even though this animal research showed knee OA after limb lengthening this is yet to be studied in humans. We evaluated physical function and the presence of radiographic signs of OA in the adjacent hip and knee joints ≥ 27 years after unilateral femoral lengthening by the callotasis technique. We hypothesized that femoral lengthening was associated with reduced physical function and OA in adjacent joints.

## Patients and methods

### Design and participants

**Figure 1.**
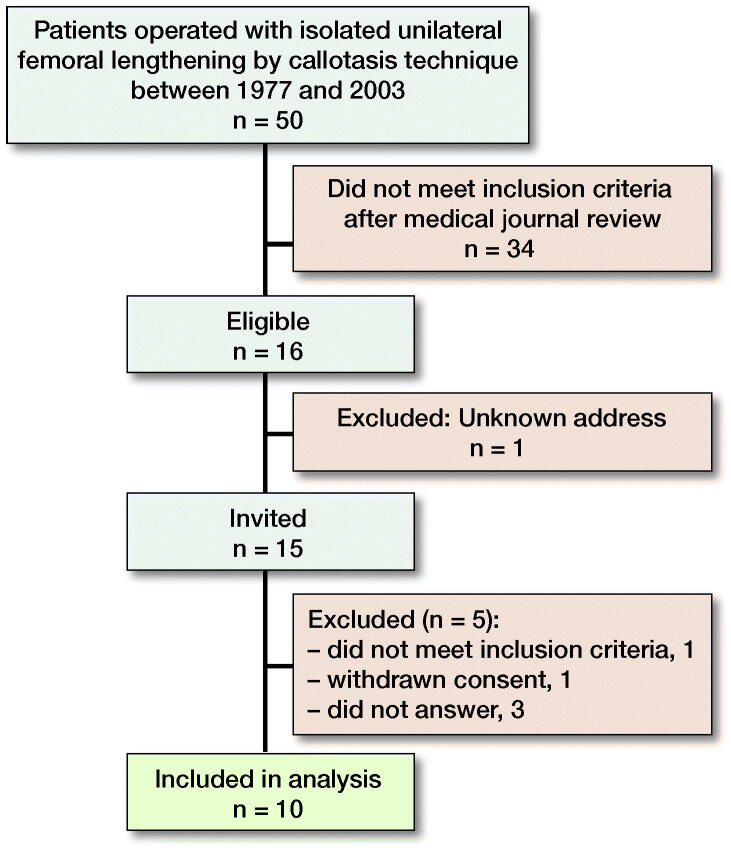
Flow chart of patients included in the study.

We conducted a cross-sectional study of 10 patients treated with unilateral femoral lengthening between 1985 and 1992. Patients were enrolled into the study during the period March–October 2019. Inclusion criteria were isolated unilateral femoral lengthening by the callotasis technique and a minimum of 15 years’ follow-up after completed lengthening—the time considered necessary to detect radiographic secondary OA changes (Schouten et al. 1992). All 50 patients who had undergone isolated femoral lengthening by the callotasis technique between 1977 and 2003 at Oslo University Hospital were identified based on protocols from the surgical department. Patients with inserted knee or hip prosthesis or any condition besides shortening that could lead to alteration of function or joint cartilage were excluded from the study. These conditions included: all cases of congenital limb shortening with concurrent axis deviation, malrotation or affection of cruciate ligaments or hip, knee, or foot deformity (proximal focal femoral deficiency/congenital femoral deficiency, fibula hemimelia, tibia hemimelia, pes equino varus). Furthermore, cases of acquired shortening with any condition that could have altered function or joint cartilage in the long term were excluded. These conditions included: hip dysplasia, Perthes disease, epiphysiolysis capitis femoris, posttraumatic shortening with joint involvement, and post-infectious shortening after septic arthritis. Patients undergoing lengthening using the Wagner method (from 1977 to 1985) were also excluded, as this method is not based on the principles of the callotasis technique. The inclusion criteria left us with cases of pure shortening due to either idiopathic leg length discrepancy, posttraumatic shortening without joint involvement either at the time of injury or at follow-up, or congenital hypoplasia or hyperplasia without axis deviation, or any pathology at the hip, knee, or foot. Following our strict inclusion criteria, 16 patients out of 50 were found to be eligible for inclusion of whom 10 consented to participate in the study (Figure 1 and Table 1, see Supplementary data).

**Table 2.** Demographic data for patients treated by unilateral femoral lengthening with callotasis (N = 10). Values are median (range) unless otherwise indicated

Characteristic	Value
Sex, female/male	7/3
Age at surgery, years	14 (13–27)
Age, years	46 (44–54)
Years between surgery and assessment	30.5 (27–34)
Height, cm	165 (157–176)
Weight, kg	75 (58–94)
BMI	28 (22–35)
Working, n	7
Living in partnership, n	8
Higher education (college/university), n	6
LLD preoperative, mm	38.5 (30–55)
Present LLD, mm	6 (–10 to 40) ^a^
Lengthened, mm	38.5 (30–57)
Days with fixator mounted	215 (135–374)
External fixator index, days/cm	52 (39–125)
Etiology, n	
Congenital LLD	
Hypoplasia	2
Hemihyperplasia	2
Developmental LLD	
Idiopathic	2
Acquired LLD	
Post traumatic	3
Sequela osteomyelitis	1

LLD = limb length discrepancy.

**^a^** Minus sign indicates overcorrection.

### Demographic data

All patients answered a set of sociodemographic questions, including age, sex, employment status, relationship status, and education level. Bodyweight and height and BMI were recorded. LLD (mm) was measured by long standing radiographs. Based on information from the medical journals, LLD was classified as either congenital, developmental, or acquired as described by Castelein and Docquier (2016). Information from the medical journals was collected to describe age at surgery, preoperative LLD (mm), preoperative alignment, the amount of lengthening (mm), and to calculate the time with fixator mounted (days), and the time from surgery to the last follow-up assessment (years). The consolidation index was expressed as the external fixator index (days with the fixator mounted per cm lengthened) (Brewster et al. 2010). Complications related to the lengthening procedure were described based on information from the medical journals. At assessment physical activity level was measured by the International Physical Activity Questionnaire short form (IPAQ short) and categorized into low, moderate, and high physical activity level according to the guidelines for the IPAQ short (IPAQ Group 2005).

### Functional tests and measurements

All patients were summoned for assessment at Oslo University Hospital. A physiotherapist (PB) did the measurements in all functional procedures.

To provide a measure of lower extremity functional strength, a 30-second sit-to-stand test (30sSTS) was conducted as described by Jones et al. (1999). To assess functional aerobic capacity, a revised stair test was performed as described by Tveter et al. (2014a), measuring submaximal cardiopulmonary endurance. Both tests are shown to be valid and reliable in patients with various musculoskeletal conditions (Tveter et al. 2014b).

To compare physical function between the lengthened and unlengthened limb, 4 single-legged hop tests were performed as described by Noyes et al. (1991) and Barber et al. (1990). The lengthened and unlengthened limb were tested twice, always starting with the unlengthened limb. The mean of 2 tests on each limb was used in the analyses. Limb symmetry index (LSI) was calculated for the single-, triple-, and cross-over hop test by dividing the mean of the lengthened limb by the mean of the unlengthened limb and multiplying the result by 100. For the timed hop test a low value (time spent) represents better performance, unlike the other 3 tests where a high value (cm hopped) is best. Hence the inverse ratio was used by dividing the mean of the unlengthened limb by the mean of the lengthened limb and the result multiplied by 100. An index of ≥ 85% has been described as normal function regardless of sports activity level, sex, and dominant side (Barber et al. 1990). The hop tests are shown to be valid and reliable in patients following anterior cruciate ligament reconstruction (Reid et al. 2007).

### Radiography

**Figure 2.**
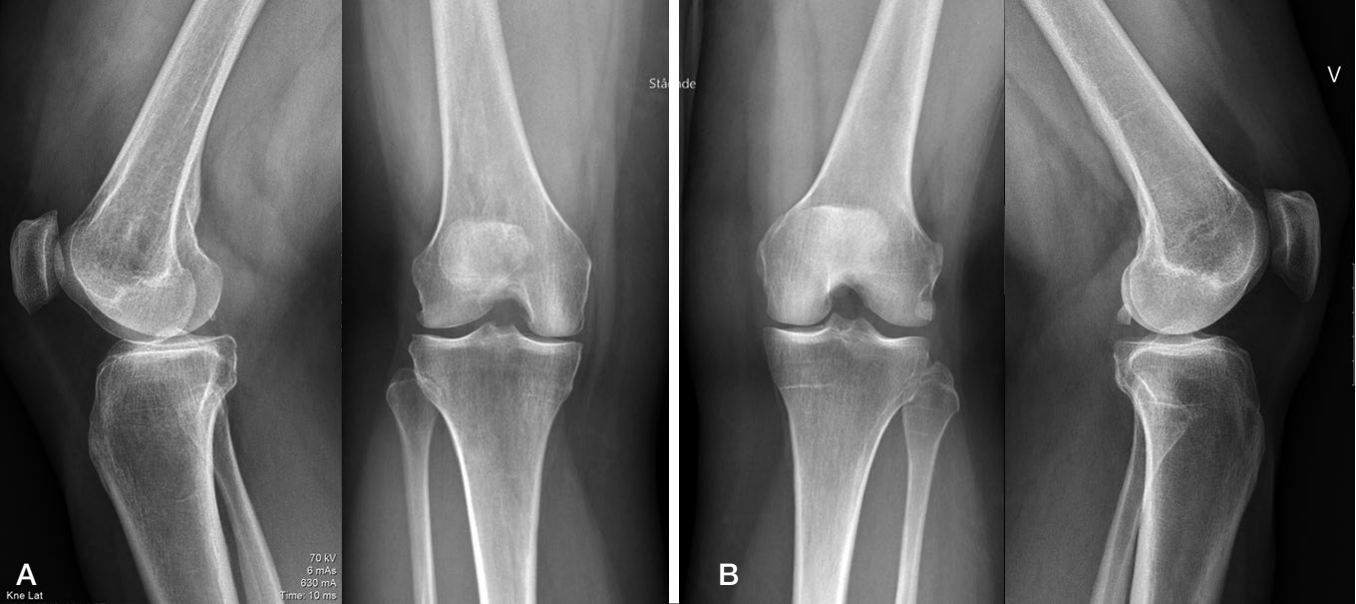
Patient number 5 in the supplementary data table. (A) The right knee with tibiofemoral and femoropatellar Kellgren and Lawrence (KL) grade 2/osteophyte. (B) The left knee with tibiofemoral KL grade 1 and femoropatellar KL grade 0.

Anteroposterior radiographs of the pelvis were taken with the patients in supine position. Film-to-focus distance was 130 cm, and all radiographs were centered 3 cm above the pubic symphysis and included the pelvis with both hips (Terjesen and Gunderson 2012). For the knees we obtained standing anteroposterior and lateral radiographs. The Syna-Flexer frame (Synarc Inc, Newark, CA, USA) was used for standardized fixed flexion positioning of the anteroposterior knee (20° flexion and 5° external foot rotation). This frame has been validated for joint space width (JSW) measurement (Kothari et al. 2004). Radiographic OA was defined as structural changes in hip and knee joints. For the hips and tibiofemoral joints OA was evaluated by measuring minimum JSW in mm and by Kellgren and Lawrence classification (KL) (Kellgren and Lawrence 1957). For the femoropatellar joint only KL was used as lateral knee radiographs are unsuited for JSW measurement. Minimum JSW was registered as the narrowest part of the upper, weight-bearing part of the hip joint and the smallest JSW of the anteroposterior knee. JSW of < 2.0 mm and KL grade 2–4 were defined as radiographic OA. For KL grading of the knees we used the precision proposed by Felson et al. (2011) dividing KL2 into KL2/osteophyte when only definite osteophyte was present, and KL2, defined as definite osteophyte and possible narrowing of joint space (Figure 2). We reported the latter only as OA. As axis deviation might occur during limb lengthening and at the same time is considered a risk factor for development of knee OA (Brouwer et al. 2007), anteroposterior and lateral long standing radiographs were taken and used to describe alignment by the zones of the mechanical axis (±1 to ±3) as described by Stevens et al. (1999). Negative zones 2–3 were classified as varus, positive zones 2–3 as valgus, and zone ±1 as normal alignment. These radiographs were taken with patella pointed forwards to eliminate rotation of the lower extremities as a source of error (Paley 2005).

### Statistics

Data were analyzed using SPSS Statistics version 26 (IBM Corp, Armonk, NY, USA). Results were presented as median (range) if continuous and frequency (%) if categorical. The paired Wilcoxon signed ranks test was used to analyze differences in performance between the patients and sex- and age-matched reference values (Tveter 2014a) for the 30sSTS and stair tests, and to analyze differences between the lengthened and unlengthened limb concerning the hop tests. Radiographic OA was presented by descriptive statistics.

### Ethics, registration, funding, and potential conflicts of interests

Ethical approval was granted by the Regional Committee for Medical and Health Research Ethics in Norway (REK South East B 2018/416, date of issue April 23, 2018) and the study is registered in ClinicalTrials.gov (NCT03966573). The patients gave their written consent before participation. This work was supported by Sophies Minde Ortopedi AS, grant number 07/2018 and 06/2019. The authors have no conflicts of interest to declare.

## Results

10 patients (7 females) were included in the study. The median time after completed lengthening was over 30 years; 9 out of 10 were in their 40s at assessment and the median lengthening was just below 40 mm (Table 2). Based on data calculated from the IPAQ short, 6 of 10 patients had low, 2 moderate, and 2 had high physical activity level at follow-up. Patients had been treated with unilateral femoral lengthening based on the callotasis technique by use of an Orthofix monolateral fixator. Preoperative long standing radiographs were not available; however, according to the medical journals 1 patient had pre-existing malalignment described as moderate knee varus, which was not operatively addressed. At assessment, a certain degree of frontal plane malalignment was found in several limbs both on the lengthened and unlengthened side (Table 3). 4 complications occurred related to the lengthening, including 2 fractures of the regenerate that were resolved by the end of treatment (1 nonoperatively treated and 1 that required a secondary procedure), 1 delayed consolidation that required bone grafting to consolidate followed by a fracture through the regenerate that resulted in in a knee varus zone -2, and 1 axis deviation allowed to heal in valgus zone +2.

**Table 3.** Alignment graded after Stevens et al. (1999) for patients treated by unilateral femoral lengthening with callotasis (N = 10)

Alignment grade	Lengthened limb	Unlengthened limb
Normal (±1)	7	8
Valgus (+2)	1	0
Varus (–2)	2	2

### Physical function

The patients scored worse on both the 30sSTS (p = 0.008) and the stair test (p = 0.007) compared with reference values (Table 4). For the stair test, the median pulse was 146 (107–173) bpm and BORG ratings of perceived exertion 14 (12–16) immediately after the test.

**Table 4.** Difference from sex- and age-matched reference values for 30 seconds sit-to-stand test (30sSTS) and stair test. Values are median (range)

Clinical field tests	Score	Difference from reference values	p-value ^a^
30sSTS, no. of repetitions	14 (12–25)	–11.5 (–17 to 0)	0.008
Stair test, s	48 (34–56)	–13 (–25 to 1)	0.007

**^a^** Wilcoxon signed ranks test.

The patients performed worse on the lengthened limb in all 4 hop tests (Table 5). However, the results were only statistically significant for the single (p = 0.005) and triple hop for distance (p = 0.007). 5 of 10 patients had impaired physical function (LSI < 85%) of the lengthened compared with the unlengthened limb on the single, cross-over, and timed hop test, and 6 of 10 on the triple hop test.

**Table 5.** Difference between the lengthened and unlengthened limb for the 4 single-legged hop tests. Values are median (range)

Single-legged hop tests	Lengthened limb	Unlengthened limb	p-value ^a^	Limb symmetry index (%) ^b^
Single, m	0.43 (0–1.0)	0.76 (0.48–1.1)	0.005	73 (0–100)
Triple, m	1.8 (0–3.5)	3.1 (2.1–3.7)	0.007	74 (0–101)
Timed, s	3.5 (0–7)	3.9 (3–5)	0.8	81 (0–112)
Cross-over, m	1.5 (0–3.2)	2.4 (1.5–2.9)	0.07	79 (0–114)

**^a^** Wilcoxon signed ranks test.

**^b^** Limb symmetry index is the median % of the performance on the unlengthened limb.

### Osteoarthritis

We found radiographic OA in the hip or knee in the lengthened limb in 3 out of 10 patients (1 hip, 2 knees) when based on JSW, and in 4 of 10 patients (2 hips, 2 knees) when based on KL. Those 2 patients with knee OA based on KL had both tibiofemoral and femoropatellar OA. The 3 patients identified by JSW also fulfilled the criteria based on KL. No patients had radiographic OA in both the hip and the knee in the lengthened limb, and no radiographic OA was found in the hip or the knee in unlengthened limbs regardless of the evaluation method. The aforementioned patient with preexisting moderate knee varus according to the medical journals showed a medial axis deviation corresponding to zone –2 on the long standing radiographs that were obtained for the current study. However, no signs of OA were found in this patient’s hips or knees at assessment.

## Discussion

Our results indicate that femoral lengthening may impair physical function in general, and/or physical function of the lengthened limb, and possibly lead to signs of radiographic OA in adjacent joints in the long term.

The results from the 30sSTS and stair test indicate a difference between the patients and reference values that extends beyond the measurement error as reported in patients with various musculoskeletal conditions (Tveter 2014b). Comparison of physical function between patients treated by femoral lengthening and age- and sex-matched reference values from a normal population has to our knowledge not previously been performed. Hence, our results are important as they indicate that femoral lengthening might lead to reduced general physical function. The patients were more sedentary and had higher BMI than the reference material, which on one hand could be a consequence of the lengthening procedure. On the other hand, we cannot rule out that the sedentary lifestyle could be random and have led to the reduced physical function without association with the lengthening procedure.

Even though the results are not unambiguous, 2 of 4 hop tests showed a statistically significant difference between the limbs and half of the patients had impaired physical function of the lengthened compared with the unlengthened limb at assessment. Our results are important as the literature is inconsistent in whether femoral lengthening impacts physical function of the lengthened limb or not (Bhave et al. 2017, Krieg et al. 2018). On one hand, we cannot rule out that the difference between the limbs was already present before the lengthening procedure as we do not have preoperative measurements. Preexisting differences have previously been described by Krieg et al. (2018), who found that the shorter limb was weaker than the longer both before and 2 years after femoral lengthening. On the other hand, the patients in the study by Krieg et al. (2018) could have had etiologies associated with impaired physical function of the lengthened limb, a weakness accounted for by the strict inclusion criteria in our study. Thus, the fact that we found impaired physical function of the lengthened limb in addition to the significant difference between the limbs in 2 of 4 hop tests strengthens the assumption of reduced physical function associated with the lengthening procedure. Our study adds to the literature suggesting that femoral lengthening might be associated with impaired physical function of the lengthened limb.

Furthermore, our results indicate a possible association between femoral lengthening and radiographic OA. The association between femoral lengthening and radiographic OA has to our knowledge not previously been described in humans. Our findings are in line with both animal research (Stanitski 1994) and assumptions in textbooks describing limb lengthening procedures (Herring 2008, Sneppen et al. 2014). However, we must acknowledge that the presence of radiographic OA in our sample could be random and explained as “natural history,” as all patients were in an age group at risk of developing OA (Sakellariou et al. 2017). In addition, we have to make reservations for the results in 1 of the patients with radiographic knee OA because of varus alignment outside the normal ranges in the lengthened limb at assessment, a known risk factor for development of knee OA (Brouwer et al. 2007). However, we believe that the absence of radiographic OA in unlengthened limbs despite the literature suggesting an association between LLD and hip OA in the longer limb (Gofton and Trueman 1971), in addition to the fact that 2 patients had varus alignment outside the normal ranges in the unlengthened limb at assessment, indicate a possible association between the lengthening procedure and development of radiographic OA in the long term.

The major limitation to our study was the small number of patients included, a consequence of our strict inclusion criteria. The patients were identified from surgical protocols containing all patients who had undergone limb lengthening at Oslo University Hospital since the first procedure in 1977, making it unlikely that any eligible patients were missing. However, restricting inclusion to patients without etiology of LLD, which may be associated with reduced physical function and OA, made it more likely that our results could be associated with the lengthening procedure itself. A further limitation was that neither the physiotherapist nor the radiologist was blinded in terms of which limb had undergone a lengthening procedure, as this could be revealed by scars after the fixator and in some cases by persisting radiological bony features due to the lengthening.

In conclusion, our results showed impaired physical function both in general and of the lengthened compared with the unlengthened limb in 10 patients treated with unilateral femoral lengthening. Additionally, our results indicate a possible association between femoral lengthening and radiographic OA in adjacent joints in the long term. When planning femoral lengthening procedures patients must be informed of the fact that we cannot rule out a possible risk of impaired physical function and development of OA in adjacent joints in the long term. However, further research is needed, and we acknowledge that, based on the small sample, we must read our results with caution without the ability to generalize.

## Supplementary data

Table 1 (general table) is available as supplementary data in the online version of this article, http://dx.doi.org/10.1080/17453674.2020.1866864
